# Moderators Influencing the Effectiveness of a Behavioral Teacher Program

**DOI:** 10.3389/fpsyg.2018.00298

**Published:** 2018-03-13

**Authors:** Betty Veenman, Marjolein Luman, Jaap Oosterlaan

**Affiliations:** Clinical Neuropsychology Section, Vrije Universiteit Amsterdam, Amsterdam, Netherlands

**Keywords:** behavioral program, teacher, ADHD, effectiveness, moderators

## Abstract

**Objective:** This study assessed which moderators influenced the effectiveness of a low-intensive behavioral teacher program for children with symptoms of Attention-Deficit/Hyperactivity Disorder (ADHD).

**Methods:** Primary school children (*N* = 114) with ADHD symptoms in the classroom were randomly assigned to the intervention program (*n* = 58; 91% male) or control group (*n* = 56; 77% male). Multilevel regression analyses assessed differential treatment gains of the intervention program in terms of ADHD symptoms and social skills. Moderators included demographic characteristics (gender, age, parental educational level), severity and comorbidity of problem behavior (ADHD symptoms, conduct and internalizing problems), social functioning, and classroom variables (teaching experience, class size).

**Results:** Results revealed larger program effects for older children and children from highly educated families and smaller beneficial effects for children with comorbid conduct or anxiety problems.

**Conclusion:** The intervention program seems more beneficial for highly educated families and children without comorbid problem behavior, but more intensive treatments appear necessary for children facing additional challenges.

**ClinicalTrials.gov registration number:** NCT02518711

## Introduction

There is a large body of evidence indicating that children with attention-deficit hyperactivity disorder (ADHD) benefit from behavioral teacher programs (for reviews, see Evans et al., 2014; Gaastra et al., 2016). However, the impact of such programs may differ, depending on, for example, characteristics of a child (Hinshaw, 2007). Identifying moderators of treatment success could assist teachers and other educational professionals to determine which students might benefit best from behavioral teacher programs.

The Positivity and Rules program is a manualized behavioral self-help program for teachers (PR program; Veenman et al., 2016) targeting ADHD symptoms in the classroom. The program does not require additional teacher training, which fosters long-term sustainability at little cost. The practical and easy-to-use manual includes a universal program involving psycho-education, classroom management and behavioral strategies (e.g., reward and time-out system), and a Daily Report Card (DRC) to target specific behavioral goals in individual children. Beneficial effects have been reported of the PR program on teacher-rated ADHD symptoms and social skills (Veenman et al., 2016). In line with meta-analytic literature indicating that positive program effects are often restrained to proximate measures (Sonuga-Barke et al., 2013), the positive program effects were not present for less-proximate measures (i.e., actigraphy, classroom observations and peer ratings; Veenman et al., 2017). Given the paucity of low-intensive behavioral teacher programs, the PR program could be a valuable contribution to existing intervention options. Therefore, the aim of this study was to identify moderators of treatment outcome of the PR program to determine which children could benefit most from the PR program.

Based on the available literature, both child and classroom characteristics were selected that might moderate the effectiveness of the PR program. Regarding demographic characteristics, the moderating effects of age and gender are worth investigating given the age-dependent decline of ADHD symptoms and conduct problems (Lahey et al., 2000; Faraone et al., 2006) and gender-specific manifestation of problem behavior, with less externalizing and more internalizing problems in girls than in boys (Gershon and Gershon, 2002). Regarding age, results are inconsistent, with some (meta-analytic) evidence suggesting that older children might profit more from (parent-based) behavioral programs (van den Hoofdakker et al., 2010; Comer et al., 2013) and other (meta-analytic) evidence indicating no moderating effect of age (Beauchaine et al., 2005; Lundahl et al., 2006; Enebrink et al., 2012; Thijssen et al., 2017). With regard to gender, results are also inconclusive. No moderating effect of gender was found in the Multimodal Treatment Study that compared the effectiveness of medication treatment, behavior management, combined treatment (medication and behavior management), and care as usual, nor in the internet-based parent program of Enebrink and colleagues (MTA Cooperative Group, 1999; Enebrink et al., 2012). However, superior treatment effects for boys with aggressive and oppositional behavior have been found in two other studies involving a behavioral classroom intervention (Good Behavior Game; Kellam et al., 2008; Witvliet et al., 2009) and in a low-intensive version of the Incredible Years behavioral parent program (Lavigne et al., 2008). An explanation for the larger program effects for boys might be that there is more room for improvement given their higher levels of externalizing behavior problems (Gershon and Gershon, 2002). Differential treatment response may also depend on parental educational level as it seems that families with limited social and economic resources are less likely to benefit from behavioral treatments (Rieppi et al., 2002; Chronis et al., 2006; Hoza et al., 2006; Lundahl et al., 2006; but see also Lavigne et al., 2008; La Greca et al., 2009; Leijten et al., 2018).

Susceptibility to behavioral treatments could also depend on severity of a child’s problem behavior at baseline. Superior treatment effects are found for children with more severe behavioral problems compared to children with less severe symptomology (Lundahl et al., 2006; Reyno and McGrath, 2006; Lavigne et al., 2008; Kolko et al., 2011; Thijssen et al., 2017; Leijten et al., 2018), although other studies report worse treatment effects for children with more severe behavioral problems (Owens et al., 2003; Van Lier et al., 2004). More room for improvement would explain why larger effects were observed for children with more severe problem behavior. However, a low-intensive behavioral self-help program such as the PR program might not be sufficient to target the specific needs of children with severe and persistent dysfunction (van den Hoofdakker et al., 2010).

Investigating the moderating effect of social functioning might also be fruitful. Literature showed limited effects of a behavioral program for children with both behavioral and social problems (Spilt et al., 2013). This seems plausible as the difficulties faced by these children might be more severe and persistent. Moreover, the perceived efficacy of teachers targeting problem behavior in children with both behavioral and social problems could be lower than children who only experience behavioral problems. Children with adequate social functioning might be more open to the introduction of new behavioral strategies in the classroom and could thus be more responsive to behavioral interventions where the focus lies on reinforcing positive behavior.

Since children with ADHD have high rates of comorbid problem behavior such as conduct problems and internalizing behavior (Angold et al., 1999), it is important to know whether comorbid problem behavior moderates the effectiveness of our teacher program. In general, studies investigating comorbidities suggest no moderating effect of comorbid ODD/CD on the effectiveness of behavioral programs (MTA Cooperative Group, 1999; Pelham and Fabiano, 2008). Other studies, however, do suggest more treatment gains for children with ADHD and high levels of internalizing problems (March et al., 2000; Van der Oord et al., 2008; van den Hoofdakker et al., 2010). Hence, differential treatment response could depend on the existence of comorbid problem behavior. Given the low intensity of the PR program, the program might be less effective for children with comorbid problem behavior who often have more functional impairments and worse long-term prognoses than children with ADHD only (Biederman et al., 1996; Connor et al., 2003; Bauermeister et al., 2007).

Finally, classroom variables such as teaching experience and class size are considered as relevant moderators. Experienced teachers are generally more confident in managing ADHD in the classroom (Reid et al., 1994), and increased teacher self-efficacy is positively related to performance (Schunk, 1984). Therefore, experienced teachers might more effectively apply behavioral and classroom management strategies in their classroom and superior treatment effects may thus be expected. Class size could also differentially affect the efficacy of behavioral interventions, because teachers perceive class size to be a barrier for managing ADHD in the classroom (Reid et al., 1994; Mulligan, 2001). In smaller classes, students receive more individual attention from the teacher and students are less likely to be distracted or disrupted by other children (Blatchford et al., 2011). The personalized approach in smaller classes might be crucial during behavioral interventions for children with ADHD.

To summarize, the current study investigated the moderating effects of diverse child characteristics and classroom variables on the beneficial effects of an 18-week self-help behavioral teacher program on teacher-rated ADHD symptoms and social skills. Based on the low intensity of the PR program, we hypothesized superior treatment effects for children of higher educated parents and for children with relatively less severe symptomology and no comorbid problem behavior. No predictions were made regarding age and gender given the highly inconsistent literature. Furthermore, children with well-developed social skills were expected to benefit more from the PR program. Finally, it was hypothesized that more teaching experience and smaller classes would foster the program’s effectiveness.

## Materials and Methods

### Participants

The study sample (*N* = 114) comprised primary school children (6–13 years) who displayed high levels of ADHD symptoms in the classroom and took part in an earlier study assessing the effectiveness of the PR program (Veenman et al., 2016). Participants were randomly assigned at school level to the intervention group (*n* = 58; 91% male) receiving the PR program (44 classrooms of 30 schools), or the waitlist control group (*n* = 56 from 43 classrooms of 34 schools; 77% male) that could receive care as usual (22% received some form of care, such as advice from a school counselor or parent training). Teachers of the participants in the waitlist control group received the PR program after having participated in this study.

Inclusion criteria were (a) high levels of teacher-rated ADHD symptoms (>90th percentile) based on the Hyperactivity/Impulsivity and/or Inattention scale of the Disruptive Behavior Disorders Rating Scale (DBDRS, Teacher version; Pelham et al., 1992; Oosterlaan et al., 2008), and (b) at least three clinical and three subthreshold ADHD symptoms on the Teacher Telephone Interview (TTI; Holmes et al., 2004), a semi-structured interview based on Diagnostic and Statistical Manual of Mental Disorders (DSM-IV; American Psychiatric Association, 2000). Exclusion criteria were (a) treatment for ADHD (including medication) at study entry or in the preceding 6 months; (b) enrollment in a daily contingency management program or another teacher program addressing behavior or social problems at study entry or in the preceding month; (c) IQ < 80 estimated using a short version of the third edition of the Wechsler Intelligence Scale for Children (WISC-III, including Block Design and Vocabulary; Sattler, 1992), or (d) a neurological or severe physical condition interfering with daily functioning. No more than two children per classroom and five classrooms per school were allowed to participate in order to limit the burden for teachers and to increase heterogeneity of teacher and school settings (Scherbaum and Ferreter, 2009). The participants’ flowchart can be found in the earlier study of Veenman et al. (2016) and reveals very low drop-out rates (3% in the intervention group and 0% in the control group, χ^2^ = 1.97, *p* = 0.16). Demographic characteristics and outcomes on screening measures are displayed in **Table 1**.

**Table 1:** Participants’ characteristics (*N* = 114) in intervention and control group.

	Intervention Group	Control Group
	(*n* = 58)	(*n* = 56)
**Demographic characteristics**		
Age (Years)	8.48 (1.85)	8.25 (1.97)
Gender (% Male)^∗^	91% (*n* = 53)	77% (*n* = 43)
IQ	104.02 (11.34)	100.21 (10.41)
Parental educational level	3.37 (0.67)	3.24 (0.95)
Race (% Caucasian)	86% (*n* = 50)	82% (*n* = 46)
ADHD diagnosis	10% (*n* = 6)	9% (*n* = 5)
Other psychiatric diagnosis	2% (*n* = 1;CD)	2% (*n* = 1;
		PDD-NOS)
**Parent DBDRS**		
Inattention	11.71 (5.23)	10.77 (5.57)
Hyperactivity/Impulsivity	11.79 (5.42)	10.47 (5.49)
ODD	5.88 (3.84)	4.93 (4.00)
CD	0.86 (1.31)	0.90 (1.40)
**Teacher DBDRS**		
Inattention	14.63 (5.26)	14.90 (5.83)
Hyperactivity/Impulsivity	15.67 (5.35)	15.03 (6.23)
ODD	6.77 (4.75)	5.95 (4.75)
CD	1.43 (1.65)	1.57 (1.90)
**TTI**		
Inattention	12.50 (6.12)	12.45 (5.34)
Hyperactivity/Impulsivity^∗^	15.62 (6.03)	12.88 (5.78)
Combined	28.12 (8.97)	25.33 (8.34)
**Outcome measures at baseline**		
ADHD symptoms (SWAN)	1.40 (0.75)	1.32 (0.68)
ADHD symptoms (SDQ ADHD)	8.26 (1.59)	7.41 (1.89)
Social skills (SSRS)	29.81 (9.89)	32.17 (7.96)

*M and *SD*s are depicted unless stated otherwise. ADHD = attention-deficit hyperactivity disorder; CD = conduct disorder; DBDRS = Disruptive Behavior Disorder Rating Scale; ODD = oppositional defiant disorder; PDD-NOS = pervasive developmental disorder-not otherwise specified; SDQ ADHD = ADHD scale of the Strengths and Difficulties Questionnaire; SSRS = Social Skills Rating Scale; SWAN = Strengths and Weaknesses of ADHD-symptoms and Normal-behavior scale; TTI = Teacher Telephone Interview (adapted with permission of SAGE publishing from Veenman et al., 2016). ^∗^*p* < 0.05.*

### Positivity and Rules Program

The PR program consists of a self-help behavioral teacher program addressing ADHD symptoms in the classroom, involving a teacher manual without additional expert training. The program involves elements of effective behavioral teacher programs (e.g., the classroom component of the Summer Treatment Program; MTA Cooperative Group, 1999) such as psycho-education for the teacher, classroom behavior management strategies (e.g., physical adjustments within the classroom, effective instructions), and contingency management (e.g., reward and time-out system; Chronis et al., 2004). These program elements are implemented by the teachers themselves in their classrooms. During the 18-week program students with ADHD symptoms and their classmates are administered a universal program encompassing basic behavior strategies used throughout the entire day (e.g., positively formulated rules, effective instructions and a reward system). Although several techniques will be familiar to most teachers, the manual instructs teachers how to systematically and adequately implement all intervention elements by providing detailed practical instructions on implementation. In addition, the PR program contains an individual program for students with ADHD symptoms, involving a DRC to reward children for achieving specific behavioral goals. The individual program consists of three intensity levels, differing in the number of times per day behavioral goals are evaluated and rewards are provided. Practical examples (e.g., examples of non-material rewards), work sheets (e.g., DRC), and flow diagrams (e.g., of the Time-out System) are included in the manual to facilitate program implementation. For a more detailed description of the PR program, see Veenman et al. (2016).

#### Implementation Fidelity

Teachers reported to have used all elements of the universal program for at least 85% of the time during the 18 intervention weeks (*M* = 0.88%; *SD* = 0.11), with the single exception of the element ‘provide the child with three compliments after a reprimand is given’ which was used on average 61% of the time (*SD* = 0.37). Most teachers (81%) reported adequate or good implementation of the universal reward system during the entire intervention, with the remaining 19% reporting inadequate implementation in 1 or 2 weeks during the course of the entire 18 weeks. Teachers reported to have used all elements of the DRC adequately most of the 18 weeks (*M* = 0.78% of the time; *SD* = 0.23). Only 15% of the teachers consulted the helpdesk during study participation.

### Outcome Measures

ADHD symptoms were assessed with the Strengths and Weaknesses of ADHD-symptoms and Normal-behavior scale (SWAN, teacher version; Swanson et al., 2006) and the ADHD scale of the Strengths and Difficulties Questionnaire (SDQ, teacher version; Van Widenfelt et al., 2003). The SWAN is an 18-item rating scale measuring the presence and severity of ADHD symptoms on a continuum. This questionnaire consists of a Hyperactive/Impulsive, Inattentive, and Combined scale, of which the average item score on the Combined scale was used as outcome measure. Items are rated on a 7-point Likert scale from -3 (far above average) to +3 (far below average). In the current study, items were reverse scored so that higher scores indicated more ADHD symptoms. The SWAN has high internal reliability (0.94–0.96) and validly assesses ADHD symptoms (Lubke et al., 2007; Young et al., 2009). The ADHD scale of the SDQ consists of five items and measures ADHD symptoms on a 3-point Likert scale (0 = not true, 1 = somewhat true, and 2 = certainly true), using the total score as outcome measure. The composite reliability of the SDQ ADHD scale is high (0.94–0.96), as well as the average variance extracted (0.77–0.82; Niclasen et al., 2013). The internal consistency of the SDQ ADHD scale (Dutch version) is also good (α = 0.89) and the concurrent validity is adequate (*r* = 0.78; Van Widenfelt et al., 2003).

To assess the child’s social skills, the Social Skills Rating Scale (SSRS, teacher version) was administered (Gresham and Elliott, 1990). The SSRS consists of 30 items divided over three subscales (Collaboration, Assertiveness and Self-Control). Items are rated on a 3-point Likert scale (0 = never, 1 = sometimes and 2 = often) and the total score was used as outcome measure. Adequate internal consistency and high predictive validity have been reported for the SSRS (κ = 0.77; Van der Oord et al., 2005).

### Moderator Variables

Moderator variables were measured prior to the start of the intervention to assess which factors moderated the effectiveness of the PR program.

#### Demographic Characteristics

The demographic characteristics used as moderators in the current study were age (in years), gender (using boys as reference group), and parental educational level. Parental educational level was measured as the average of both parents’ educational level using an adapted version of the Dutch educational classification system (1 = primary education, 2 = secondary vocational education, 3 = secondary general education, 4 = undergraduate school, 5 = graduate school; Verhage, 1983).

#### Severity of Problem Behavior

Severity of ADHD symptoms at baseline was measured using the SWAN (see section “Outcome Measures”) and severity of conduct problems and internalizing problems at baseline was assessed with the SDQ (measuring conduct problems with the Conduct scale, and internalizing problems as the sum of the Emotional and Peer Problems scale). Each SDQ scale (Van Widenfelt et al., 2003) consists of 5 items on a 3-point Likert scale (0 = not true, 1 = somewhat true and 2 = certainly true). The internal consistency of the scales is adequate (0.74 < α < 0.76), as well as the composite reliability (0.88–0.96) and average variance extracted (0.73–0.85; Niclasen et al., 2013). Self-reported anxiety at baseline was measured using the Spence Children’s Anxiety Scale (SCAS; Spence, 1997). The SCAS consists of 44 items, which are scored on a 4-point Likert scale (0 = never, 1 = sometimes, 2 = often, and 3 = always). The total score was used as moderator variable. The reliability of the SCAS is high (Cronbach’s α = 0.92 and McDonald’s omega = 0.95) and validity is also well established (Spence, 1998; Nauta et al., 2004).

#### Social Functioning

The moderators related to social functioning were teacher-reported social skills as measured by the SSRS (see section “Outcome Measures”) and peer acceptance. Peer acceptance was measured through peer ratings, in which participants and their classmates were asked to indicate how much they liked each classmate on a 5-point rating scale (1 = dislike very much, 3 = neutral, 5 = like very much; Hoza et al., 2005). Adequate reliability and validity have been reported for peer ratings (*r* = 0.47 and *r* = 0.44; Olsen et al., 2001). Social preference was used as indication of peer acceptance and was calculated following the procedure of Maassen and colleagues (Maassen et al., 1996; Maassen et al., 2000).

#### Comorbid Problem Behavior

Moderating effects of comorbid problem behaviors (conduct problems, internalizing problems, anxiety problems and limited social skills) were assessed using the same instruments as described above. Hence, the SDQ (teacher version) was used to measure conduct problems and internalizing problems, the SCAS as measure of self-reported anxiety and the Teacher SSRS and peer ratings as measures of social skills.

#### Classroom Variables

The moderating classroom variables used in current study were teaching experience (in years) and class size (i.e., the number of students in each class).

### Procedure

This study was conducted in the Netherlands between September 2011 and July 2014. Teachers and parents were recruited through educational consultant associations, the national parent association for children with developmental problems, and the study’s website. In case teachers were interested in participating, they enlisted one or two students displaying ADHD symptoms in their classroom. Written informed consent was obtained from all parents, teachers, and participants older than 11 years. Participants were screened for eligibility by the first author BV. ML, who had not been in contact with any participants, was responsible for the subsequent computer-generated randomization to allocate children to the intervention or control group. Teachers were aware of treatment allocation given their involvement in treatment delivery. Parents and children were also aware of treatment allocation, as they were aware of the obvious classroom changes (e.g., the rewarding system for the entire classroom and the use of the DRC). Although the universal program was used in the entire classroom, only children displaying ADHD symptoms in the classroom were included as participants in this study. Dependent variables were measured at three time points: at baseline (T_o_), 6 weeks after start of the PR program (T_1_), and after 18 weeks at the end of the intervention (T_2_). For the children in the intervention group, the SWAN was also administered in week 9, 12, and 15 after start of the intervention to monitor progress and to adjust the intensity level of the individual program (see Veenman et al., 2016). For all moderating variables, baseline scores were used. All participating teachers received a small financial compensation (control group: €50; intervention group: €125). This study was carried out in accordance with the recommendations of the medical ethical committee of the Vrije Universiteit Amsterdam with written informed consent from all subjects. All subjects gave written informed consent in accordance with the Declaration of Helsinki. The protocol was approved by the medical ethical committee of the Vrije Universiteit Amsterdam (reference number 2011/196).

### Statistical Analyses

To assess which factors moderate the effectiveness of the PR program, multilevel regression analyses were conducted using the Statistical Package for the Social Sciences (SPSS; IBM Corp, 2011). All randomized subjects participated in the intention-to-treat analyses, regardless of the amount of missing data. For all measures, the percentage of missing data was low (3.6% for the SWAN; 2.6% for the SCAS, and 1.8% for the SDQ, SSRS and Peer Rating). Missing data were not imputed as multilevel analyses adequately deal with missing data (Twisk et al., 2013).

Four hierarchical levels were used: observations were nested within students, students were nested within classrooms, and classrooms were nested within schools (level 4; Heck et al., 2013). First, the overall intervention effects on ADHD symptoms (measured by the SWAN and SDQ ADHD scale) and social skills (measured by the SSRS) were estimated by using Group as dichotomous independent variable, while controlling for baseline levels of the dependent variable. Subsequently, separate multilevel regression analyses were conducted to assess the moderating effects of the demographic characteristics (gender, age, and parental educational level), severity of problem behavior (ADHD symptoms, conduct problems, internalizing problems, and anxiety), social functioning (teacher-reported social skills and peer acceptance), and classroom variables (teaching experience and class size). This was first done by centering all moderators (except for gender), after which the moderator and interaction between group and moderator were added to the original model that consisted of Group and baseline level of the dependent variable.

The moderating effects of comorbid problem behaviors (conduct problems, internalizing problems, anxiety problems, and social skills) were assessed by looking at three-way interactions between group and all possible pairs of comorbid problem behaviors (e.g., group × ADHD symptoms × conduct problems; group × ADHD symptoms × anxiety symptoms; etcetera). Similar three-way interactions were used to assess whether the level of social skills in combination with other problem behaviors (ADHD symptoms, conduct problems, internalizing problems, and anxiety) moderated treatment gains on social skills. In case of significant three-way interactions, *post hoc* analyses were performed to assess treatment effects for four different groups, using a median-split for both variables (e.g., anxiety and ADHD symptoms). If an interaction involved the SWAN ADHD scale (measuring ADHD symptoms), the term ‘relatively high’ and ‘relatively low’ levels of ADHD symptoms were used to describe children scoring at one of the extreme ends of the ADHD continuum. Alpha-level was set at 0.05.

## Results

**FIGURE 1:**
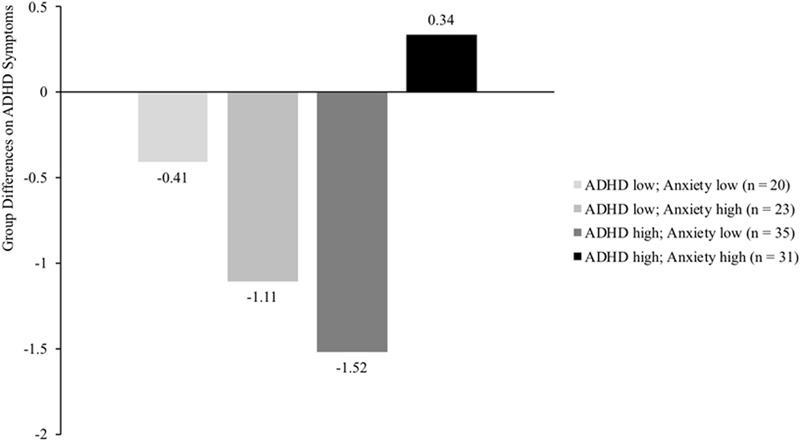
Differences between intervention and control group on ADHD symptoms (measured with SDQ ADHD scale) for four groups of children differing in level of ADHD symptoms (median split SDQ ADHD: score > 7 indicates high levels of ADHD symptoms) and anxiety problems (median split SCAS: score > 18 indicates high levels of anxiety problems). Results demonstrate a significant reduction of ADHD symptoms for intervention children scoring high on ADHD symptoms and low on anxiety (*p* = 0.013), compared to children in the control group. No significant group differences were found for children scoring high on both moderators or low on both moderators (*p* = 0.603 and *p* = 0.597, respectively), nor for children scoring relatively low on ADHD symptoms but high on anxiety (*p* = 0.063).

**FIGURE 2:**
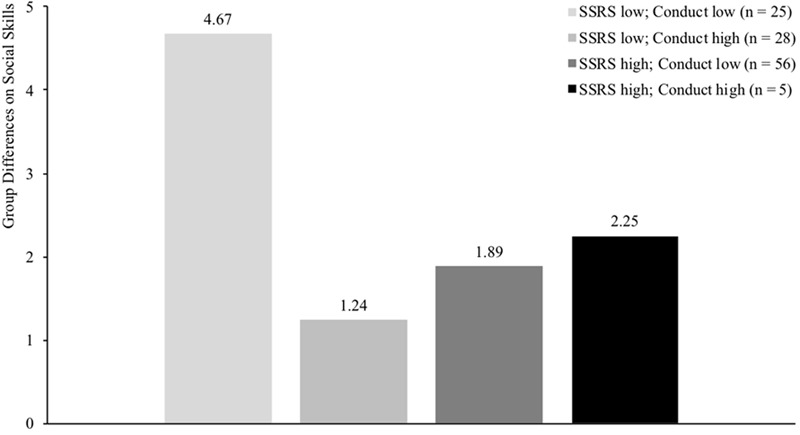
Differences between intervention and control group on social skills (measured with SSRS) for four groups of children differing in level of social functioning (median split SSRS: score > 31 indicates high levels of social functioning) and conduct problems (median split SDQ: score > 1 indicates high levels of conduct problems). SSRS = Social Skills Rating Scale. Results demonstrate largest treatment gains for children scoring low on both social skills and conduct problems (*p* = 0.018) compared to the other groups where no significant differences were found (*p*-values > 0.26).

Results of the multilevel regression analyses, assessing what moderators influenced the effectiveness of the PR program, are displayed in **Table 2**. The three-way interactions assessing the moderating effects of the program are not displayed in **Table 2**, but the significant three-way interactions are visualized in **Figures 1** and **2**.

**Table 2:** Results of moderation multilevel analyses.

	SWAN ADHD		SDQ ADHD		Social skills	
Moderating effects	Coefficient (*SE*)	*p*-value	Coefficient (*SE*)	*p*-value	Coefficient (*SE*)	*p*-value
**Demographic characteristics**						
Age	-0.12 (0.05)	0.036^∗^	-0.14 (0.19)	0.459	0.95 (0.60)	0.119
Gender	-0.16 (0.25)	0.528	-0.31 (0.97)	0.749	-0.84 (2.97)	0.779
Parental educational level	-0.37 (0.10)	<0.001^∗∗^	-0.45 (0.44)	0.844	2.35 (1.31)	0.076
**Severity of problem behavior at baseline**						
ADHD symptoms (T)	0.12 (0.13)	0.341	-0.08 (0.19)	0.664	-	-
Conduct problems (T)	0.03 (0.05)	0.472	0.00 (0.17)	0.981	-	-
Internalizing problems (T)	0.02 (0.03)	0.456	-0.04 (0.11)	0.730	-	-
Anxiety (Self-Report)	0.009 (0.006)	0.138	0.00 (0.03)	0.929	-	-
**Social functioning at baseline**						
Social skills (T)	-	-	-	-	-0.09 (0.11)	0.426
Peer acceptance	-	-	-	-	-2.47 (0.87)	0.005^∗∗^
**Classroom variables**						
Experience teacher	0.01 (0.01)	0.186	0.03 (0.04)	0.430	-0.16 (0.10)	0.117
Class size	-0.02 (0.03)	0.499	-0.08 (0.09)	0.362	0.16 (0.28)	0.567

*Tabulated effects on teacher-rated ADHD symptoms (SWAN and SDQ ADHD) and social skills pertain to interactions between the moderator of interest and group (intervention group versus control group). The other fixed effects and all random effects are not displayed in this Table, but can be obtained upon request. Lower scores on the SWAN and SDQ ADHD indicate improvement (i.e., reduction) of ADHD symptoms. Higher scores on social skills indicate improvement (i.e., increase) of social skills. SDQ = Strengths and Difficulties Questionnaire; SWAN = Strengths and Weaknesses of ADHD-symptoms and Normal Behavior. T = teacher rating. *^∗^ p* < 0.05. ^∗∗^*p* < 0.01.*

### Moderators for Intervention Effects on ADHD Symptoms

Before assessing which moderators influenced the effects of the PR program on ADHD symptoms as measured by the SWAN and the SDQ ADHD scale, primary analyses confirmed positive intervention effects on ADHD symptoms when controlling for baseline ADHD symptoms (*b*(*SE*) = -0.50(0.11), *p* < 0.001 for the SWAN and *b*(*SE*) = -0.87(0.35), *p* = 0.017 for the SDQ ADHD). With regard to the SWAN, moderating effects were found for Age and Parental educational level, indicating that the intervention effect on ADHD symptoms was more beneficial for older compared to younger children (*b*(*SE*) = -0.12(0.05), *p* = 0.036) and for children from highly educated families compared to lower educated families (*b*(*SE*) = -0.37(0.10), *p* < 0.001). No significant moderating effects were found for any of the other variables on ADHD symptoms as measured by the SWAN.

Results on the SDQ ADHD did not reveal a significant moderating effect for Age nor for Parental educational level. The SDQ results did reveal a significant moderating effect for comorbid anxiety problems, suggesting smaller program effects for children with high levels of both ADHD symptoms and self-reported anxiety (*b*(*SE*) = 0.04(0.02), *p* = 0.014). To illustrate this interaction, *post hoc* analyses were conducted to assess treatment effects for four different groups (relatively low ADHD symptoms and low anxiety, relatively high ADHD symptoms and high anxiety, or one of the two variables high and the other variable low). Results of these *post hoc* analyses (see **Figure 1**) suggest that children with relatively high levels of ADHD symptoms (>7 on the SDQ ADHD scale that ranged from 0 to 10) and high levels of anxiety problems (>18 on the SCAS that ranged from 1 to 78) did not profit from the PR program in terms of ADHD symptoms (*b*(*SE*) = 0.34(0.63), *p* = 0.603). Significant treatment gains were found for children with relatively high levels of ADHD symptoms but low levels of anxiety (*b*(*SE*) = -1.52(0.56), *p* = 0.013), meaning a reduction of almost one standard deviation on the SDQ ADHD scale (*M* = 7.85; *SD* = 1.79). No significant treatment effects were found for the other two groups (children scoring relatively low on ADHD symptoms but high on anxiety, *p* = 0.063, and for children scoring low on both variables, *p* = 0.597). No other significant moderating effects were found on ADHD symptoms as measured by the SDQ ADHD.

### Moderators for Intervention Effects on Social Skills

Before assessing the moderating effects on social skills, primary analyses confirmed that that social skills of children in the intervention group significantly improved compared to children in the control group when controlling for baseline social skills (*b*(*SE*) = 3.26(1.26), *p* = 0.003). Results revealed a significant moderating effect for peer acceptance (*b*(*SE*) = -2.47(0.87), *p* = 0.005), indicating that the intervention was more effective for children who were less accepted by peers at baseline. Furthermore, results also showed a higher effectiveness of the PR program for children with low levels of both conduct problems and teacher-rated social skills (*b*(*SE*) = 0.16(0.06), *p* = 0.014). This finding was confirmed by *post hoc* analyses assessing treatment effects for four different groups (low SSRS and low conduct, high SSRS and high conduct, or one of the two variables high and the other low) by dichotomizing both variables through median split (>31 for the SSRS and >1 for the SDQ Conduct scale). *Post hoc* results showed largest treatment gains for children scoring low on both social skills and conduct problems (*b*(*SE*) = 4.67(1.85), *p* = 0.018) compared to the other groups (*p*-values > 0.26; see **Figure 2**). This gain for children scoring low on both social skills and conduct problems indicated that social skills of these children improved approximately half of one standard deviation on the SSRS (*M* = 30.95; *SD* = 9.07). No other significant moderating effects were found on social skills.

## Discussion

This study aimed to investigate which child and classroom variables moderated the effects on teacher-rated ADHD symptoms and social functioning of the PR program, a behavioral teacher program targeting ADHD symptoms in the classroom. Significant moderating effects were found for age and parental educational level on ADHD symptoms (as measured by the SWAN), indicating that the effect of the PR program on ADHD symptoms was larger for older children and for children from highly educated families. Results on the SDQ ADHD measure suggest larger treatment gains for children with both high levels of ADHD symptoms and low levels of anxiety, while program effects appeared absent for children with high levels of both ADHD symptoms and anxiety. The effects of the PR program on social functioning was significantly moderated by peer acceptance, indicating that the intervention was more effective for children who were less accepted by peers at baseline, and by the interaction between social skills and conduct problems, indicating superior program effects for children who scored low on social functioning and did not have serious conduct problems.

This study isolated what type of children would benefit most from our self-help behavioral teacher program. Results show that our intervention might be more effective for older children, for children from highly educated families, and for children with high levels of ADHD symptoms in the absence of other problem behavior (i.e., conduct problems or anxiety). These findings are in line our hypothesis that this low-intensive behavioral teacher program could be more effective for children from highly educated families, and those without many comorbid psychopathology. For children from low educated families and for those with severe comorbid problem behavior, more intensive therapeutic support might be needed to successfully reduce ADHD symptoms and improve social functioning (van den Hoofdakker et al., 2010).

Regarding the moderating role of age, our results are in line with (meta-analytic) evidence that also revealed larger effects of behavioral programs for older children (van den Hoofdakker et al., 2010; Comer et al., 2013) although there is also meta-analytic evidence revealing no moderating effects of age (Lundahl et al., 2006). Perhaps, older children can more easily inhibit their impulses and comply with the challenges imposed by teachers implementing the PR program than younger children, because brain development and socialization improves with age (Faraone et al., 2006).

The absence of a moderating effect of gender is in line with the MTA study where gender did not moderate treatment outcomes either (MTA Cooperative Group, 1999), and suggests that the PR program is equally effective for boys and girls. However, it could also be that no moderating gender effect was found due to the high percentage of boys participating in our study (84%), resulting in limited power to detect gender differences. Indeed, moderating effects of gender have been found in other studies where roughly half of all participants were girls (see for example Lavigne et al., 2008; Witvliet et al., 2009). More research on samples with a more equal gender distribution is necessary to confirm whether the PR program is equally effective for both boys and girls.

Our finding that the PR program is more effective for children from highly educated parents is in line with several reviews discussing predictors and moderators of treatment outcomes in children with ADHD (Chronis et al., 2006; Hoza et al., 2006). In most of those treatments, however, parents were involved in delivery of treatments (e.g., medication and parent programs), in which case the moderating role of parental educational level could be explained by greater involvement and greater treatment adherence of highly educated parents (La Greca et al., 2009). This explanation is less likely in our study where parents were not involved in treatment delivery. A more likely explanation might be related to teachers’ attitudes and expectations. Children from families with a low socioeconomic status (SES, often quantified by parental education) are often perceived less positively by teachers and teachers have lower expectations of these children in terms of academic and behavioral functioning (McLoyd, 1998). Hence, it is possible that teachers also expect less treatment improvement in these children.

The current results indicate that the PR program is equally effective for all children with ADHD symptoms, regardless of the severity of their ADHD symptoms, which is in line with meta-analytic evidence (Lundahl et al., 2006; Reyno and McGrath, 2006). However, children with comorbid conduct or anxiety symptoms do seem to profit less from this program. More specifically, no treatment gains on ADHD symptoms were found for anxious children with high levels of ADHD symptoms, while large improvements on ADHD symptoms were found for non-anxious children with high levels of ADHD symptoms. The reason that others did find larger treatment gains for behavioral programs in children with ADHD and comorbid anxiety (March et al., 2000; Van der Oord et al., 2008; van den Hoofdakker et al., 2010) could be related to the larger involvement of parents in these programs. Parents of children with ADHD and comorbid anxiety symptoms could be more anxious and overprotective toward their child than parents of children without comorbid anxiety symptoms (Pfiffner and McBurnett, 2006) and might thus be more inclined to conform to treatment protocols (Van der Oord et al., 2008), possibly resulting in larger treatment gains. In this manualized teacher program, parents were not involved in treatment delivery at all, which could explain the different results of this study. Perhaps, the perceptions of teachers can explain the lower gains of the PR program for anxious participants. Anxious children are likely to show very intense help-seeking and proximity-seeking behaviors toward teachers, which could result in teachers perceiving the child as challenging and causing frustration (Pianta and Nimetz, 1991), thus impeding treatment gains. Providing teachers with more elaborate information on comorbid internalizing and externalizing in children with ADHD and additional therapeutic support might help to increase the effectiveness of the PR program (Gillberg et al., 2004).

For children scoring low on social functioning, the PR program appears to be more effective in improving teacher-rated social skills, particularly if conduct problems are absent or not very severe. Possibly, children with limited social skills who do not display oppositional or aggressive behavior (e.g., children who are shy or withdrawn) have more room to improve their social functioning thanks to enhanced classroom structure and positive reinforcement provided by the PR program. In contrast, children with both limited social skills and conduct problems, might be more likely to oppose the changes made in the classroom during the implementation of the PR program, which could thus result in smaller benefits for those children.

The current findings need to be interpreted in light of the following limitations. First, power was somewhat limited due to the relatively small sample size, urging cautious interpretation of the current findings. Second, our sample mainly comprised boys, limiting the reliability of the finding that the PR program would be equally effective for boys and girls. This problem is common in ADHD research though, where boys are clearly overrepresented in the diagnosed ADHD population (Gershon and Gershon, 2002). Third, we acknowledge that we did not assess the influence of other potentially interesting variables on treatment outcome, such as problems at home (e.g., maternal depression or marital problems) or several teacher variables (e.g., teachers’ treatment expectations, treatment acceptance, and compliance). Fourth, this study did not investigate the influence of non-specific treatment effects on treatment outcomes such as positive treatment expectations. Future research is thus necessary to distinguish the specific from non-specific treatment effects.

### Summary

This study investigated the moderating effects of several child and classroom variables on the effectiveness of a low-level behavioral teacher program (PR program) in children with ADHD symptoms. Results indicated that the PR program could be more effective for older children and for children from higher educated families, while comorbid conduct or anxiety problems in combination with high levels of ADHD symptoms appeared to impede treatment gains. Hence, it appears that this self-help teacher program is suitable for children not facing additional challenges, but may not be able to address the needs of children from low educated families and those with comorbid psychopathology. The current findings underline the importance of using the PR program in a preemptive stage before behavioral problems escalate or transfer to multiple domains.

## Author Contributions

BV: data collection, statistical analyses, literature review, and writing entire manuscript. ML and JO: article revisions.

## Conflict of Interest Statement

The authors declare that the research was conducted in the absence of any commercial or financial relationships that could be construed as a potential conflict of interest.
